# How accurately do behavioural observations predict reproductive success in free-ranging lizards?

**DOI:** 10.1098/rsbl.2019.0030

**Published:** 2019-02-27

**Authors:** Mats Olsson, Tonia S. Schwartz, Erik Wapstra, Richard Shine

**Affiliations:** 1 Department of Biological and Environmental Sciences, University of Gothenburg, Medicinaregatan 18, 413-90 Gothenburg, Sweden; 2 Department of Biological Sciences, Auburn University, Auburn, AL 36849, USA; 3 School of Biological Sciences, University of Tasmania, Private Bag 55, Hobart 7001, Australia; 4 School of Life and Environmental Sciences, University of Sydney, Sydney, New South Wales 2006, Australia

**Keywords:** fitness, Lacertidae, reproductive output, reptile, Sweden

## Abstract

Behavioural ecologists often use data on patterns of male–female association to infer reproductive success of free-ranging animals. For example, a male seen with several females during the mating season is predicted to father more offspring than a male not seen with any females. We explored the putative correlation between this behaviour and actual paternity (as revealed by microsatellite data) from a long-term study on sand lizards (*Lacerta agilis*), including behavioural observations of 574 adult males and 289 adult females, and paternity assignment of more than 2500 offspring during 1998–2007. The number of males that contributed paternity to a female's clutch was correlated with the number of males seen accompanying her in the field, but not with the number of copulation scars on her body. The number of females that a male accompanied in the field predicted the number of females with whom he fathered offspring, and his annual reproductive success (number of progeny). Although behavioural data explained less than one-third of total variance in reproductive success, our analysis supports the utility of behavioural-ecology studies for predicting paternity in free-ranging reptiles.

## 1. Introduction

To test ideas about the adaptive significance of mating systems, we need to measure the impact of behavioural variation on individual fitness. For females, we can measure the production of progeny to obtain a measure of annual reproductive success; but for males, the challenge is greater because paternity of offspring is uncertain, especially in internally fertilizing species [1]. Copulations are difficult to observe in the field, and (even if observed) may not lead to paternity of offspring. For example, the female partner may fail to reproduce, or may use sperm from another male when she does so, or the resultant embryo may die before hatching from the egg or before intact DNA can be harvested for paternity assignment.

Most scientific literature on mating systems in reptiles perforce has relied upon behavioural data, with correlates of reproductive success in males identified from traits such as numbers of copulations, or numbers of females with whom a male is seen in close proximity [2–4]. Molecular methods to establish paternity have been used to clarify mating systems of an increasing number of reptilian taxa [5–9], but few studies have gathered the data needed to compare male behaviour with paternity. We have such data for a population of lizards, and the present paper compares behaviourally based indicators of male reproductive success with measures based on molecular analysis of paternity for more than 2500 hatchlings.

## 2. Methods

### (a) Study species and area

Sand lizards (*Lacerta agilis*) are diurnal surface-active lacer-tids with a broad geographical distribution [10,11]. The population we studied at Asketunnan in Sweden (57°22′ N, 11°59′ E) inhabits a rocky archipelago site surrounded by the ocean [12–14] close to the northern limit of the species' range [10]. The lizards are active above ground from March each year, mate in April through to early June and females lay a single clutch of eggs in June [12,13]. Clutch size averages around nine eggs (range 5–15: [15]). Males mate-guard females after copulation, and we often see male–female pairs in close association at this time [16,17]. Females mate with multiple males, but selectively use sperm from distantly related males to fertilize their eggs [18,19].

### (b) Field methods

Throughout the mating season over the period 1998–2007, we visited the study site on as many days as possible when weather conditions were suitable for lizard activity (see [12–14] for details). We recorded male–female associations, and collected tissue samples from all adult lizards for use in paternity analyses (see below). We collected females when their bodily distension suggested that egg-laying was imminent, and returned them to the laboratory, where they were maintained until oviposition. Eggs were incubated in the laboratory, and hatchlings were released at the study site after tissue samples had been taken (see [19] for detailed methods).

### (c) Laboratory methods

We conducted parentage analysis using cervus v.3.0 [20] based on 17–21 microsatellites resulting in a non-exclusion probability of 5.87×10^−5^ with one parent known (details available in [21]; see also the electronic supplementary material). In brief, DNA was isolated from 4543 adult and offspring samples (blood and tissue) collected over a 9-year period (1998–2006), representing 3938 individuals. Our analysis was based on the subset of these animals for which we had complete data on parental traits in our mating system analyses. Because of the low level of genetic variability in this population and the overlap of generations, it was necessary to use 17–21 microsatellite loci to assign paternity with high confidence [21].

### (d) Statistical analyses

We used ANOVA (in JMP v.13.1; SAS, Cary, NC, USA) to conduct the following analyses, using individual animals within each year as the unit of replication. For data on females, we used the number of males with whom a female had progeny per year as the dependent variable, and either the number of males a female was seen with in the field in that year or the number of copulation scars (left by the jaws of males during mating) as independent variables. Some females were recorded in multiple years, so we included female ID and year as random factors in these analyses.

For males, the independent variable was the number of females with whom a male was seen during the mating season. Our dependent variables were either total number of progeny per annum (as determined by paternity analyses), or the number of clutches (females) to which a male contributed paternity per year. Male ID and year were included as random factors. We conducted these male-specific analyses on two datasets: one consisting of all adult-size males (greater than 60 mm snout–vent length) and one consisting only of males that were recorded to father offspring in the year in question.

## 3. Results

Our analyses below are based on data for 289 female lizards that were each present in the field population for a mean of 1.64 years as reproducing adults. In total, those females produced 3626 offspring (mean = 12.67, range 1–55 per female), of which we were able to assign paternity to 2384 ( = 66%). We also obtained behavioural data on 252 males that were successful in obtaining paternity (in a mean of 1.76 years each, range = 1–7 years), and 322 that sired no offspring (i.e. had zero reproductive success). The analysis including all adult males was based on 574 individuals, which were present in the dataset for a mean of 1.70 years (range 1–8 years) per male.

### (a) Females

**Figure 1.**
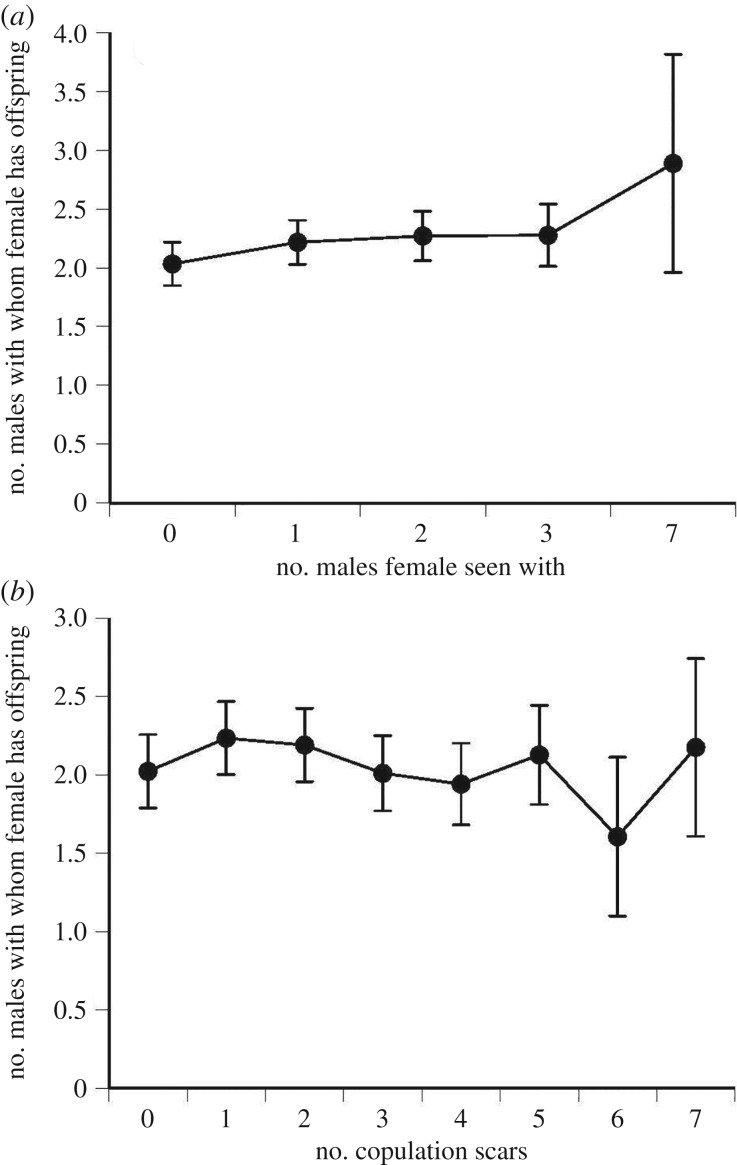
The number of males contributing paternity to a female sand lizard's clutch as a function of (*a*) the number of males she was seen with in the field during the mating season and (*b*) the number of copulation scars on her flanks.

The number of males that fathered a female's progeny in any given year was positively correlated with the number of males with which she was seen in the field (*F*_1,369.8_ = 4.158, *p* = 0.0422, *r*^2^ = 0.33) but not significantly correlated with the number of copulation scars that we counted on her flanks (*F*_1,322_ = 0.44, *p* = 0.51, *r*^2^ = 0.36; figure 1).

### (b) Males

**Figure 2.**
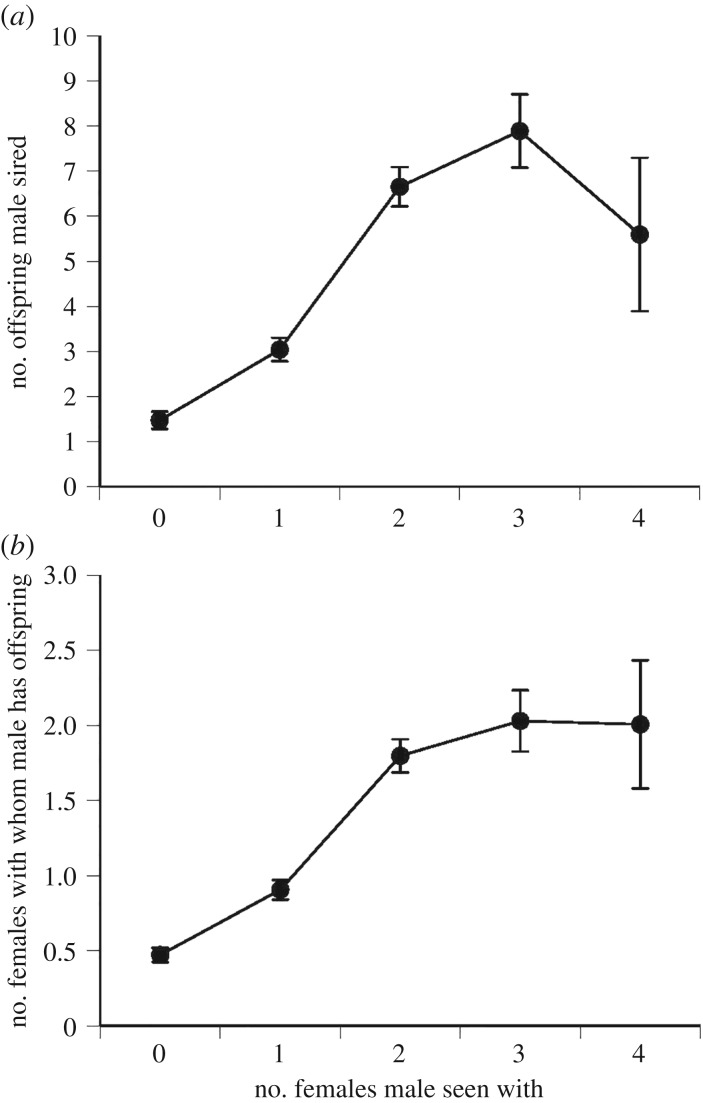
The number of female sand lizards with which a male was seen in the field as a function of (*a*) the number of offspring he sired and (*b*) the number of females to whose clutches he contributed paternity.

Within the subset of males that were reproductively successful in a given year, the number of females a male was seen with was positively correlated with his reproductive success (number of offspring: *F*_1,211.6_ = 18.84, *p* < 0.0001, *r*^2^ = 0.13) and with the number of females with which he had offspring (*F*_1,214_ = 23.20, *p* < 0.0001, *r*^2^ = 0.12). The same patterns were evident, but stronger, if the analysis included all males rather than only the reproductively successful ones (number of offspring: *F*_1,707.1_ = 153.17, *p* < 0.0001, *r*^2^ = 0.20; number of females with which a male had offspring: *F*_1,707.1_ = 167.23, *p* < 0.0001, *r*^2^ = 0.22; figure 2).

## 4. Discussion

Our analysis provides empirical support for a critical but rarely tested assumption of research on the behavioural ecology of reptiles: that an individual's reproductive success can be inferred from indirect measures based on the animal's behaviour. Significant correlations between microsatellite-determined paternity and behavioural traits (male–female proximity, home range size, aggregation) have been reported from field studies of scincid, agamid and xantusiid lizards [7,22,23]. An extensive literature documents multiple paternity within natural clutches of many reptile species [24,25], sometimes associated with behavioural traits (e.g. mating order [26]). In our population of sand lizards, knowledge of male–female associations in the field predicted the extent of multiple paternity within clutches, and also a male's total reproductive success (in terms of number of offspring as well as number of clutches to which he contributed paternity). However, correlations between behavioural variables and actual paternity were relatively low (explaining only 12–36% of variance in paternity). Surprisingly, the number of copulation scars evident on a female lizard (widely used as a proxy for the number of times she has copulated [25,26]) was not significantly correlated with the number of males fathering her offspring.

Overall, our results are both encouraging (simple-to-record behaviours are indeed associated with male reproductive success) and discouraging (correlations between behaviour and paternity are relatively low). The relationship between the two sets of scores tended to be higher in males than in females, especially if non-successful males were included in the analysis. The only clearly non-significant result was the lack of association between the number of copulation scars on a female versus the number of fathers of the eggs in her clutch. That result may reflect rapid healing of scars, such that earlier copulations fail to be scored when the female is collected late in the mating season. Also, a male may mate more than once with a female, leaving multiple mating scars [25,26]. Our results suggest that fieldworkers should interpret copulatory scars with care.

In our study population (and likely, in many others), the link between matings and paternity is weakened by non-random use of sperm by females [18], as well as by random ‘noise’ in the data. For example, we may have failed to observe some male–female pairings because they were brief, or occurred in places or at times when we failed to note the animals. Likewise, progeny from some pairings may have been inviable (and hence never scored for paternity), for example, owing to genetic incompatibility between partners resulting in mortality occurring so early in embryonic development that we were unable to obtain viable DNA for molecular analysis [18]. Given the array of such potential confounding effects, the significant predictive value of male–female association data for inferring male reproductive success and multiple paternity within clutches is reassuring. The degree to which behavioural data predict genetic measures of reproductive success will depend upon a range of factors specific to study species and systems. For example, male sand lizards mate-guard females for long periods, increasing the investigators' ability to detect male–female associations during the mating season. Technological advances doubtless will make paternity assessment increasingly easier and cheaper; but our data suggest that even in the absence of such molecular analyses, behavioural-ecology studies can provide robust insights into the correlates of variance in male reproductive success in free-ranging reptiles.

## Supplementary Material

Microsatellite paternity assignment_Supplementary

## Data accessibility

Data are available via Dryad: http://doi.org/10.5061/dryad.73d9482 [27].
