# Supplementary material for: How accurately do behavioural observations predict reproductive success in free-ranging lizards?
Source: Biol Lett. 2019 Feb 27;15(2):20190030. doi: 10.1098/rsbl.2019.0030 (PMC6405472; doi:10.1098/rsbl.2019.0030)
Supplement: Microsatellite paternity assignment_Supplementary [file rsbl20190030supp1.docx]

**Microsatellite paternity assignment**

**Genotyping**

DNA was isolated from 4,543 adult and offspring samples (blood and tissue) collected over a nine year period (1998-2006), representing 3,938 individuals (Table 1). Of these, we entered individuals into our analyses for which we had complete data on parental traits in our mating system analyses. Samples were genotyped using 21 microsatellite loci: LA01, LA02, LA03E, LA04, LA10, LA27, LA37, LA40, LA45, LA47, LA50, LA55, LA58 (Schwartz & Olsson 2008); LA1-AG, LA2-AG, LA3-AG (Gullberg et al. 1997); Lvir7, Lvir17 (Bohme et al. 2005); and two loci (LV4-72 and LV4-X) (Boudjemadi et al. 1999) for which primers were redesigned to optimize multiplexing (Table 1). The 21 microsatellite loci were amplified in multiplexed PCR reactions each containing 3-6 loci, using fluorescently labeled primers. Multiplexed PCR reactions were run in a 7 μl volume and contained 250-850 pmol of each primer, 0.22 mM dNTPs, 1.1X Qiagen PCR buffer with 1.5mm MgCl2, additional MgCl2 when necessary (Table 2), 0.03 U of hot start *Taq* (Qiagen), and 10-50 ng DNA. Thermal cycling conditions had a 15 min hot start at 95°C, 30 cycles of 95°C for 20 sec, 52-56°C annealing for 30 sec, 72C for 1.15 minutes, and a final extension at 72°C for 30 minutes to three hours. Sample electrophoresis was run on an ABI 3130xl genetic analyzer and analyzed in GeneMapper and corrected by eye (Applied Biosystems).

**Paternity Analysis**

Because of the low level of genetic variability in this population (Gullberg et al. 1997; Table 1), and the overlap of generations, it was necessary to use 17-21 microsatellite loci to assign paternity with high confidence. All of the adults and the offspring from years 2001-2006 were genotyped at 21 loci. The offspring from years 1998-2000 were genotyped with 17 loci except for offspring for which paternity between the two top candidate males could not be determine with 95% confidence; these offspring were genotyped at all 21 loci. Paternity analyses were conducted in Cervus 3.0 (Kalinowski et al. 2007) for the nine years of data 1998-2006. A four-year sliding window of adult genotypes was used to calculate the population allele frequencies for each year of offspring analyzed. Genotyping error rate in the final data set is estimated to be less than one percent based on mother-offspring comparisons, repeated genotyping of the same individuals collected in multiple years, and from 30% of the data being independently scored by a second researcher. Paternity was assigned based on two simulation analyses, complete exclusion, and 1% error rate (Table 2). Candidate sires included all adult males sampled in previous years, the year the offspring were born, and the subsequent year. Confidence levels of Delta (the difference between the LOD scores of the first and second most likely candidates) were set at 95% and 80%. The paternity assignment was classified using three “confidence codes” based on the type of data that supported the assignment. (1) 95% confidence level of the mother-offspring-father Delta based on either the zero error rate or the 1% error rate simulation. (2) 80% confidence level of the mother-offspring-father Delta, and additional evidence of partnership from field observations (witnessing copulations or mate guarding between the assigned sire and the mother). (3) 80% confidence level of the mother-offspring-father Delta and evidence of the male contributing to the clutch due to siblings being independently assigned the same father with at least 80% confidence. If paternity could not be assigned based these confidence codes, the individual was eliminated from subsequent analyses.

**Paternity Analysis - Field Born Adults**

Since we have multi-generational data, many of the field born adults (FBA) that were sampled each year are likely to be offspring from adults sampled the same or previous years. Thus, parentage analyses were conducted on all field born adults to identify potential mother-father pairs. Parentage analyses were conducted as based on a simulation of 1000 offspring, a zero error rate (complete exclusion), and assuming 85% of candidate mothers and fathers sampled. All adults in the population were assumed to be potential parents. Parents identified with 95% confidence level of Delta were cross-checked with all available data on age, size, and field location of the potential parents relative to the individual of interest to ensure the identified parental pairings were possible.

**Paternity Assignment**

We had high confidence in our ability to assign a parent if the parent had been sampled. Across years, the average probability of non-exclusion of a candidate parent if neither parent was known was 0.006, the average probability of non-exclusion of a candidate parent if one parent was known was 5.87E-05. In total, 2,757 offspring were successfully assigned a father (Table 3). On average 89% of the assignments had both a 95% confidence level of Delta in Cervus and additional evidence from either field observations or clutch information supporting the paternity assignment.

Gullberg, A., H. Tegelström, and M. Olsson. 1997. Microsatellites in the sand lizard (*Lacerta agilis*): Description, variation, inheritance, and applicability. Biochem. Gen. 35:281-295.

Boudjemadi, K., Martin, O., Simon, J. C. and A. Estoup. 1999. Development and cross-species comparison of microsatellite markers in two lizard species, *Lacerta vivipara* and *Podarcis muralis*. Mol. Ecol. 8:518-520.

Kalinowski, S. T., Taper, M. L. and T. C. Marshall. 2007. Revising how the computer program Cervus accommodates genotyping error increases success in paternity assignment. Mol. Ecol*.* 16: 1099-1106.

Table 1 (Could be supplementary table or referenced): Details of microsatellite loci used for parentage analysis. Size range of alleles (Size), number of alleles (N), polymorphic information content (PIC), and the observed heterozygosity H_O_, based on all the entire dataset of 3967 genotyped individuals.

| Locus | Fluorophore-Primer sequence 5’-3’ | PCR ^f^ | Size | N | PIC | H_O_ |
| --- | --- | --- | --- | --- | --- | --- |
| LA01 ^a^ | VIC-AACGGAGGTAGAATGTCATAGC  CTTGAAGGGAAAGAGCTACTGC | Mplx1 | 110-120 | 6 | 0.351 | 0.372 |
| LA02 ^a^ | FAM-TGCCTGCAAGACTATAATCCAAG  GGAATGGCATGAGATATGGTG | Mplx1 | 222-244 | 8 | 0.719 | 0.736 |
| LA03E ^a^ | VIC-AAAGTTGGTCTGCACTGACG  CAATTCAAAATGCACACAACG | Mplx2 | 243-245 | 2 | 0.098 | 0.099 |
| LA04 ^a^ | VIC-CTAGGCATGGAGAATGGATGTG  AGCCACTTCCCTAAGTGTGTCC | Mplx3 | 141-191 | 6 | 0.357 | 0.434 |
| LA10 ^a e^ | VIC-TAATAAAGCAGGCGCAAACC  TGTTGTGTGGAATTGTGAGC | Mplx5 | 175-225 | 8 | 0.700 | 0.731 |
| LA27 ^a^ | ATCTGGCGGAGGGATGAG  VIC-AAATGCAAGCGAGCAACAAT | Mplx1 | 137-155 | 9 | 0.403 | 0.432 |
| LA37 ^a^ | CCACCCCATTCTCATAAAAGG  PET-TTTGCTTGGAGCTTCTGTCC | Mplx3 | 110-128 | 2 | 0.208 | 0.237 |
| LA40 ^a^ | GGGAACCGTTGTACTAAGTTTGG  VIC-ATGCATTCAGATGTCTCCCAAG | Mplx1 | 191-203 | 5 | 0.556 | 0.615 |
| LA45 ^a^ | NED-CAGAGTTCATGGAAAGTGAAGG  AAGGAGACTCTGCTGGTCATTC | Mplx2 | 203-221 | 6 | 0.520 | 0.555 |
| LA47 ^a^ | PET-CCCACTAGAGAAATGAGCTTCTG  CAAACAAGGAGGGTAAGGAATG | Mplx2 | 111-127 | 7 | 0.543 | 0.623 |
| LA50 ^a^ | FAM-AGGTAGCCCAGGTGTCATACAG  TGGGTCTTACATGAGCTGAATC | Mplx2 | 109-123 | 6 | 0.594 | 0.638 |
| LA55 ^a^ | NED_TCCCTCATTACAGGCATAGGAG  TCTGAACAAAACATGGGACTTG | Mplx1 | 126-144 | 5 | 0.552 | 0.626 |
| LA58 ^a^ | FAM-CAGTTCTGGGGATTTTCTCCTAC  CATTGTAATTGGAGCACAAAGC | Mplx2 | 165-179 | 6 | 0.575 | 0.645 |
| LA64 ^a^  \ | PET-AGATGCTGAACTACCAGCTTGC  GCTATCCTGGCTGACCATTAAG | Mplx3 | 185-192 | 3 | 0.277 | 0.348 |
| LA1-KB | FAM-AGGTTTCCTGGCTTGGAG  ATTTGCACAAAACAGCAGC | Mplx3 | 101-135 | 15 | 0.821 | 0.843 |
| LA2-AG ^b e^ | GCTTAAATTGGAACCAGATTG  FAM-AAGCAGCCAGAACACAGAG | Mplx5 | 187-193 | 4 | 0.351 | 0.39 |
| LA3-AG ^b^ | AGTAGGAGCGAGAAGAATCAG  NED-GACATATGGCAGAAAGAGCAG | Mplx4 | 158-188 | 8 | 0.739 | 0.786 |
| LV4-72 ^c^ | NED-CAAAGCCAAAGAAGGCTCTC  CTTTGCAGGTAACAGAGTAGTTC | Mplx4 | 97-113 | 5 | 0.668 | 0.717 |
| LV-4-X ^c^ | PET-TGAAACATGGATTAGAGGCTGA  ACTCCTTGCGTGGCATAAAA | Mplx4 | 165-172 | 6 | 0.323 | 0.352 |
| Lvir7 ^d e^ | VIC-TCGACAGCTTGCAGGCTTGAC  GAAGGGCTCTTCCAGACACTG | Mplx5 | 320-360 | 8 | 0.732 | 0.779 |
| Lvir17 ^d e^ | FAM-AGCTCTGGATCGAGACAACCTGG  TCTCTGAAGGAGACCGGCTCC | Mplx5 | 312-334 | 5 | 0.628 | 0.666 |

^a^ Schwartz & Olsson 2008.

^b^ Gullberg et al. 1997.

^c^ newly designed from Boudjemadi et al. 1999.

^d^ Bohme et al. 2005.

^e^ additional 4 loci included in the 21-locus genotypes

^f^ PCR conditions: MPLX1: 56ºC annealing; 2.75 mm MgCl_2_. MPLX2: 56ºC annealing; 3.15 mm MgCl_2_. MPLX3: 56ºC annealing; 2.65 mm MgCl_2._ MPLX4: 52ºC annealing; 3.15 mm MgCl_2_. MPLX5: 52ºC annealing; 2.15 mm MgCl_2_

Table 2: Data sets used in Cervus for paternity analysis. Allele Freq; individuals used to calculate population allele frequencies. Candidate Fathers; male individuals that were included as potential candidate fathers. Prop Males: proportion of candidate males in the population assumed to have been sampled. Assign. Rate: expected paternity assignment rates assuming the mother is know, based on simulations of either zero exclusion, or assuming a 0.01 error rate. A = adults, JR = released juveniles released that were recaptured.

| Offspring  Year | Allele Freq  Dataset | Candidate  Fathers Dataset | No. Candidate Fathers | Prop  Males | Assign. rate  Exclusion | Assign. rate  Error 0.01 |
| --- | --- | --- | --- | --- | --- | --- |
| 1998 | A 1998-2000 | A 1998-2000 | 133 | 0.8 | 82% | 76% |
| 1999 | A 1998-2000 | A 1998-2000  JR 1998 | 149 | 0.9 | 72% | 86% |
| 2000 | A 1998-2001 | A 1998-2001  JR 1998-1999 | 194 | 0.9 | 89% | 81% |
| 2001 | A 1999-2002 | A 1998-2002  JR 1998-2000 | 244 | 0.9 | 91% | 88% |
| 2002 | A 2000-2003 | A 1998-2003  JR 1998-2001 | 282 | 0.9 | 90% | 93% |
| 2003 | A 2001-2004 | A 1998-2004  JR 1998-2002 | 366 | 0.9 | 90% | 92% |
| 2004 | A 2002-2005 | A 1998-2005  JR 1998-2003 | 413 | 0.9 | 91% | 90% |
| 2005 | A 2003-2006 | A 1998-2006  JR 1998-2004 | 470 | 0.9 | 91% | 91% |

Table 3. Summary of success in paternity analysis by year and confidence codes. (1) 95% confidence level of the mother-offspring-father Delta based on either the zero error rate or the 0.01% error rate simulation. (2) 80% confidence level of the mother-offspring-father Delta, and additional evidence of paternity from field observations (witnessing copulations between the male and the mother or mate guarding, etc). (3) 80% confidence level of the mother-offspring-father Delta and evidence of the male contributing to the clutch due to siblings being independently assigned the same father with at least 80% confidence. Multiple evidence is the percent of individuals that had 95% confidence in Cervus and other evidence, either field observations or other siblings with independent assignment of same father, supporting the possibility of a correct paternity assignment. FBA are field born adults.

| Year | Total  offspring | %  assigned | (1) | (1)(2) | (1)(2)(3) | (1)(3) | **any**  **(1)** | (2) | (2)(3) | **any**  **(2)** | (3) | Total  assigned | Multiple evidence |
| --- | --- | --- | --- | --- | --- | --- | --- | --- | --- | --- | --- | --- | --- |
| 1998 | 296 | 67 | 5 | 2 | 116 | 64 | **187** | 0 | 6 | **6** | 6 | 199 | 91% |
| 1999 | 399 | 59 | 10 | 7 | 97 | 100 | **214** | 0 | 14 | **14** | 6 | 234 | 87% |
| 2000 | 304 | 76 | 34 | 15 | 97 | 70 | **216** | 0 | 12 | **12** | 3 | 231 | 79% |
| 2001 | 333 | 87 | 11 | 4 | 166 | 86 | **267** | 0 | 13 | **13** | 8 | 288 | 89% |
| 2002 | 485 | 83 | 27 | 12 | 183 | 171 | **393** | 0 | 6 | **6** | 3 | 402 | 91% |
| 2003 | 430 | 93 | 23 | 6 | 118 | 245 | **392** | 0 | 4 | **4** | 5 | 401 | 92% |
| 2004 | 428 | 91 | 38 | 6 | 47 | 290 | **381** | 0 | 2 | **2** | 5 | 388 | 88% |
| 2005 | 509 | 82 | 29 | 3 | 125 | 247 | **404** | 0 | 4 | **4** | 7 | 415 | 90% |
| 2006 | 267 | 75 | 12 | 0 | 21 | 163 | **196** | 0 | 1 | **1** | 2 | 199 | 92% |
| **Total** | **3451** | **713** | **189** | **55** | **970** | **1436** | **2650** | **0** | **62** | **62** | **45** | **2757** |  |
| **avg** | 383.3 | 79.2 |  |  |  |  |  |  |  |  |  |  | 89% |
| FBA | 517 | 38 | 45 |  |  |  |  |  |  |  |  | 45 |  |
